# Developing nephrology services in low income countries: a case of Tanzania

**DOI:** 10.1186/s12882-019-1568-7

**Published:** 2019-10-17

**Authors:** Francis F. Furia, Jacqueline Shoo, Paschal J. Ruggajo, Kajiru Kilonzo, Gopal Basu, Karen Yeates, Santosh Varughese, Einar Svarstad, Onesmo Kisanga

**Affiliations:** 1 School of Medicine, Muhimbili University of Health Sciences (MUHAS), P. O. Box 65001, Dar es Salaam, Tanzania; 2 Renal Unit, Muhimbili National Hospital (MNH), Dar es Salaam, Tanzania; 3 Department of Internal Medicine, Kilimanjaro Christian Medical Centre (KCMC), Moshi, Tanzania; 4 Department of Renal Medicine, The Alfred Hospital, Melbourne, Australia; 5 Previously Department of Nephrology, Christian Medical College, Vellore, Tamil Nadu India; 6 Department of Medicine, Division of Nephrology, Queen’s University, Kingston, Ontario Canada; 7 Department of Nephrology, Christian Medical College-Vellore, Vellore, Tamil Nadu India; 8 Department of Medicine, Haukeland University Hospital and Department of Clinical Medicine, University of Bergen, Bergen, Norway

**Keywords:** Nephrology in Tanzania, Nephrology training, Nephrology in sub-Saharan Africa

## Abstract

**Background:**

The burden of kidney diseases is reported to be higher in lower- and middle-income countries as compared to developed countries, and countries in sub-Saharan Africa are reported to be most affected. Health systems in most sub-Sahara African countries have limited capacity in the form of trained and skilled health care providers, diagnostic support, equipment and policies to provide nephrology services. Several initiatives have been implemented to support establishment of these services.

**Methods:**

This is a situation analysis to examine the nephrology services in Tanzania. It was conducted by interviewing key personnel in institutions providing nephrology services aiming at describing available services and international collaborators supporting nephrology services.

**Results:**

Tanzania is a low-income country in Sub-Saharan Africa with a population of more than 55 million that has seen remarkable improvement in the provision of nephrology services and these include increase in the number of nephrologists to 14 in 2018 from one in 2006, increase in number of dialysis units from one unit (0.03 unit per million) before 2007 to 28 units (0.5 units per million) in 2018 and improved diagnostic services with introduction of nephropathology services. Government of Tanzania has been providing kidney transplantation services by funding referral of donor and recipients abroad and has now introduced local transplantation services in two hospitals. There have been strong international collaborators who have supported nephrology services and establishment of nephrology training in Tanzania.

**Conclusion:**

Tanzania has seen remarkable achievement in provision of nephrology services and provides an interesting model to be used in supporting nephrology services in low income countries.

## Background

Non-communicable diseases (NCD) are reported to contribute significantly to mortality and morbidity globally, with more than 70% of global mortality estimated to be attributable to NCD [1, 2]. Tanzania, like many other sub-Saharan African (SSA) countries is faced with a growing burden of non-communicable diseases. The rising burden of NCD has put strain on the health system which historically was overwhelmed by managing communicable diseases [3]. Kidney diseases have contributed significantly to increased risk and burden of cardiovascular diseases as well as mortality in Tanzania and other low and middle-income countries (LMICs) [4].

There is global disparity in the availability of nephrology services with limited or non-existing services in many lower-income countries most of which are in sub-Saharan Africa. Resources needed for addressing the growing burden of chronic kidney disease (CKD) in this region are scarce; these include diagnostic and therapeutic equipment as well as the human resources and facilities for training them [5]. A few countries in sub-Saharan Africa have managed to establish some form of services; these services are of limited scope and not comprehensive enough to address the huge burden of CKD in the region [6].

Global efforts have seen the establishment and improvement of nephrology services in many lower income countries, and the International Society of Nephrology (ISN) has played a significant role in training nephrologists [7, 8]. Despite these efforts there is still a huge gap in the availability and access to these services in most of sub-Saharan Africa [9].

Tanzania located in East Africa and with a population of over 55 million people is divided administratively into 30 regions and 7 zones. Health system in Tanzania is organized into clearly defined referral system linking primary health care facilities to the national hospital. Dispensary which serve an estimated population of 10,000 people is the lowest level and it feeds patients into health centres which has capacity of emergency surgeries. Several health centres are supported by the district hospital. Each region has one regional hospital and 4–6 district hospitals, regional hospitals refer patients to zonal consultant hospitals and the national hospital. There are four zonal consultant hospitals all of which offer specialized services. There are several private hospitals most of which are in big cities which also provide specialized services.

Tanzania has made significant improvement in the provision of services for non-communicable diseases in the past two decades. Among the NCD which has high burden is diabetes mellitus, in 2006 about 400,000 people were estimated to be diabetic therefore Tanzania Diabetes Association and ministry of health established diabetic clinics in many regional hospitals [10]. This progress has been made possible through collaborative support from several international institutions and organizations. This article is aimed at highlighting the current status of renal diseases and nephrology services in Tanzania. The strategies, key achievements and current challenges facing the national health system in provision of nephrology services are discussed. The importance of international support and collaborative initiatives are outlined, and the Tanzanian experience could potentially serve as a model for implementing modern nephrology services in resource limited settings.

### Burden of kidney diseases in Tanzania

There is paucity of data on the burden of kidney diseases in Tanzania as is the case in many resource-limited countries. Available administrative and research data from hospital and community-based facilities indicate a high prevalence of kidney diseases. Stanifer et al. reported a CKD prevalence of 7% in the community setting in Northern Tanzania, with significantly higher prevalence in urban setting (15%) as compared to rural setting (2%) [11]. Ploth et al. documented a CKD prevalence of 12.4% while that of hypertension and diabetes were 19.9 and 14.8% respectively in rural Eastern Tanzania, advanced CKD was noted to be higher among young people aged 18–36 years [12].

Hospital based reports have also documented a high burden of kidney diseases. Janmohamed et al. reported a CKD prevalence of 83.7% among 397 diabetic patients attending a diabetic clinic, out of which 24.3% had eGFR of less than 60 ml/min from a study conducted at Bugando Medical Centre, a consultant zonal hospital in North-western Tanzania [13]. Another study from the same hospital reported renal dysfunction in 27.5% out of 637 adult patients admitted to the medical wards between October 2013 and March 2014 [14]. .A study conducted at the emergency medical department at Muhimbili National Hospital (MNH) between September 2017 and February 2018 reported renal failure in 8.8% out of 3013 screened patients, out of which 71 and 195 had acute kidney injury (AKI) and CKD respectively and 146 had indications for dialysis [15].

Human Immunodeficiency virus (HIV) infection, whose burden is high in sub-Saharan Africa, with a prevalence of 5% in Tanzania, is an important risk factor for kidney diseases in Tanzania. Msango et al. reported a prevalence of severe renal dysfunction (eGFR 30–59) of 24.5% out of 355 patients starting anti-retroviral therapy [16].

Kayange et al. documented a prevalence of kidney dysfunction (eGFR < 60 ml/min) of 7.4 and 4.9% among HIV infected and un-infected children respectively, in a study conducted among admitted children at Bugando Medical Centre [17]. Fredrick et al. also reported a prevalence of severe renal dysfunction (eGFR 30–59 ml/min) of 5.8% in a study conducted among HIV infected children attending clinic at Muhimbili National Hospital [18].

## Methodology

This situation analysis undertaken to determine provision of nephrology services in Tanzania, which was approved by MUHAS institutional Review Committee, was carried out by interviewing key personnel in the facilities providing nephrology services in Tanzania. No individual information was sought during interviews therefore a waiver of consent was also granted by MUHAS IRB. Interviews were carried out through phone calls using a checklist which collected information about number of dialysis patients, number of haemodialysis machines in each unit, international collaborators and support provided by each international collaborator. Information about individual nephrologist/nephropathologist training was obtained from Nephrology Society of Tanzania. Information about kidney transplantation was obtained from Muhimbili National Hospital and Benjamin Mkapa Hospital which are currently offering local kidney transplantation services. Muhimbili National Hospital which coordinated overseas kidney transplantation provided information transplant services obtained out of Tanzania.

## Results

### Nephrology services

Several services are offered for kidney diseases in Tanzania, and there has been a rapid increase in the number of facilities offering services for treatment of patients with end-stage kidney disease (ESKD). These services include haemodialysis, peritoneal dialysis and kidney transplantation. Importantly, renal clinics offering diagnostic services have been established in four zonal consultant hospitals which serve and average of 4–8 million population for each zone. Nephrologists and other clinicians provide services in renal clinics. Diagnostic supports for nephrology services to guide management of patients have been established including nephropathology [19].

### Haemodialysis

Table 1 describes the location, number of dialysis machines and patients on maintenance haemodialysis in Tanzania as of 31st December 2018. There are 28 haemodialysis centres (0.5 units/million), some of these are in hospitals while others are stand-alone dialysis centres equipped with medical laboratory, procedures and consultations rooms. Most of the dialysis centres 75% (21/28) are privately owned and located in large cities in seven regions (23.3%) out of all 30 regions in Tanzania. Fifteen (53.6%) out of all dialysis units are in Dar es Salaam city. Both acute and maintenance haemodialysis are offered in most of the dialysis centres, patients with AKI are referred to stand alone dialysis units from hospitals not offering dialysis services [20].

**Table 1:** Distribution of dialysis units in Tanzania

Name of Region (Estimated population)	Name of Dialysis unit	Number of Haemodialysis machines	Number of CRRT^e^ machines	Number of patients on dialysis
Dar es Salaam (4,364,541 million)	Muhimbili National Hospital^a^	42	1	227
	Muhimbili National Hospital- Mloganzila^a^	12		29
	Agha Khan Hospital^b^	8		21
	Emilio Mzena Hospital^a^	6		8
	TMJ Hospital^c^	7		40
	TMJ polyclinic^c^	5		3
	Regency Medical Centre^c^	22		120
	Al-Shifaa Clinic^c^	8		17
	Hindu Mandal Hospital (Dar es Salaam)^b^	8		35
	Hindu Mandal (Kunduchi)^b^	10		9
	Kairuki Hospital^c^	7	1	27
	Access Health Centre^c^	16		84
	Sali International Hospital^c^	1		NO^d^
	Apollo polyclinic^c^	4		NO^d^
	CCBRT^b^	8		NO^d^
Arusha (1,694,310 million)	Arusha Lutheran Medical centre^b^	6		33
	NSK Hospitals^c^	13		51
Kilimanjaro (1,640,087 million)	Kilimanjaro Christian Medical Centre^b^	5		31
Dodoma (2,083,588 million)	University of Dodoma Health centre^a^	10		21
	Benjamin Mkapa Hospital^a^	10	1	26
Mbeya (2,707,410 million)	Mbeya Referral Hospital^a^	5		20
	Afya Check^c^	4		7
Mwanza (2,772,509 million)	Bugando Medical centre ^b^	10		60
	Access Medical Centre^c^	8		4
	Bio Health Centre^c^	5		10
	Hindu Mandal^b^	10		NO
Zanzibar (1,303,569 million)	Mnazimmoja Hospital^a^	6		34
	Global Hospital^c^	3		16
TOTAL		259	3	933

^a^Public institution; ^b^Private not for profit including faith-based institutions; ^c^Private institution

^d^Not operational; ^e^Continuous Renal Replacement Therapy

### Peritoneal dialysis

Acute peritoneal dialysis (PD) has been offered in Tanzania since 2009. This program was implemented at Kilimanjaro Christian Medical Centre (KCMC) and initially established and supported by the Sustainable Kidney Care Foundation (SKCF) (New York, USA) in partnership with the International Paediatric Nephrology Association (IPNA) and the ISN. After the program was established, the Saving Young Lives (SYL) program supported by ISN was introduced and continued to support the program at KCMC through support for training of health providers to provide acute PD. This program began with capacity building among doctors and nurses who were trained in Curitiba, Brazil. Initial supplies of catheters and PD fluids were provided through the SKCF [21–23]. Bugando Medical Centre is another centre in Tanzania, which has received support from SYL program for the establishment of acute PD services. At Muhimbili National Hospital, PD was introduced in 2012, and has been offered largely for children with acute kidney injury [24], and recently Agha Khan Hospital in Dar es Salaam has introduced acute peritoneal dialysis for children with AKI.

### Kidney transplantation

Kidney transplantation services were initiated for Tanzanians since the 80’s. Initially end-stage kidney disease patients and their donors had to travel overseas for services. The first batch of patients who received kidney transplantation services were sent to St Thomas Hospital in United Kingdom [25]. Thereafter, patients were being sent to several hospitals in India, and a small number were sent to other countries including Kenya. A total of 250 patients had received kidney transplantation surgery overseas (mostly in India) with government funding of transplant programs which covered air travel and living expenses for donors and recipients. These patients and their donors received pre-transplantation work up at Muhimbili National Hospital (MNH) which included extensive evaluation of both donors and recipients prior to referral. Only living related donors whose relationship had been verified by DNA profiling could donate under this program, in order to curtail possibility of commercial unrelated donor kidney transplantation. After surgery, the pair would stay near the transplant centre for 3–6 months for initial post-transplantation care and then continue with follow up care at MNH.

In November 2017 Tanzania started offering kidney transplantation services locally. These have been introduced in two hospitals, MNH and Benjamin Mkapa Hospital (BMH) in Dodoma, the country’s capital city. These services have been made possible by collaborating with hospitals from India and Japan for MNH and BMH respectively. Transplant surgeons, nephrologists, anaesthesiologists and nurses from India and Japan paired with local teams and carried out transplant surgeries and provided immediate post-transplant surgery care in this program. With ongoing capacity building that seeks to improve local capacity, the dependence on the visiting team continues to decline. A total of 28 patients have undergone transplantation since November 2017, out of which 24 were performed at MNH and 4 at BMH. Currently, the national transplant program only includes living related donor kidney transplants.

### Kidney biopsies

Parallel with development in renal replacement therapies described above, the nephrology trainees received training in doing kidney biopsy in Haukeland University Hospital, Norway and Christian Medical College, Vellore, India. The 1st kidney biopsy (in modern era) was conducted in 2012 at Muhimbili National Hospital and since then more than 100 renal biopsies including renal graft biopsies have been performed using modern real time ultrasound-guided techniques. The biopsies taken have aided in determining the underlying diagnoses and have guided both therapy and prognosis. The Haukeland University Hospital has sustained the kidney biopsy-based nephrology practice in Tanzania by promoting hands-on-ultrasound-guided kidney biopsying through training as well as donating the starter-up gear (GE Logic 7 Ultrasound Machine, 2 Spring Loaded Magnum Bard Biopsy Guns and several kidney biopsy needles among others). The training of a Tanzanian nephropathologist in Norway, under the ISN Fellowship, on interpretation of both native kidney biopsies and allograft histopathology has added value to service development in the region. Regency Medical Centre is another hospital offering renal biopsy services in Tanzania.

### Human resources

The number of nephrologists has increased in the past decade in Tanzania, from one in 2006 to 14 nephrologists in 2018. Nephrologists in Tanzania have been trained through a foreign government supported program to train local nephrologists introduced in 2007 at Muhimbili University of Health and Allied Sciences (MUHAS) as well as through the ISN fellowship program [26]. Ten nephrologists have been trained in the locally established training program at MUHAS and two of these nephrologists were through local partnerships with African countries including Ethiopia and Uganda.

The MUHAS Nephrology training program was initially offered in collaboration with University of Bergen in Norway and Christian Medical College in Vellore, Tamil Nadu- India. Candidates were initially trained in all three institutions and received their qualifications from MUHAS. Currently there are six nephrology trainees, three are training at MUHAS and three are under the ISN fellowship program with two in South Africa and one in India. Nephrology training at MUHAS is a 2 years program admitting physicians with training in internal medicine or general paediatrics, the program is housed in the department of Internal Medicine under School of Medicine.

MUHAS has been offering pathology training program at postgraduate level, this program had limited exposure in nephropathology until recently following introduction of nephropathology at Muhimbili National Hospital after successful completion of ISN fellowship in nephropathology by one pathologist from Tanzania. Some of pathology trainees at MUHAS, including the ISN fellow in nephropathology, have been participating in ISN-ANIO CNC online courses which have equipped them with a strong foundation and interest in nephropathology [27].

Nurses have largely provided haemodialysis care for ESKD patients in Tanzania. Training has occurred through programs where nurses receive short-term training (2-3 months) overseas; with most going to India and some to Pakistan. Currently dialysis nurses are receiving training for few weeks to months in well-established dialysis units (mainly at the Muhimbili National Hospital). In a few centres, qualified dialysis technicians who are foreign nationals provide haemodialysis services.

In order to address the shortage of renal nurses, Muhimbili University of Health and Allied Sciences is in the final process of establishing post-graduate training in renal nursing (Master of Science in Renal nursing). This program will have four semesters for a duration of 2 years and will enrol nurses with a bachelor’s degree. Table 2 shows the numbers and the countries in which nephrologists and nephropathologist have received training.

**Table 2:** Institutions and countries in which existing Tanzanian nephrologists and nephropathologist were trained

Country of training	Number of trained doctors	Discipline	Training institution	Qualification
Tanzania	5^a^	Nephrology	MUHAS, UiB (Norway), Haukeland University Hospital (Norway), Christian Medical College, Vellore (India)	MSc Nephrology
	5	Nephrology	MUHAS (with external rotation at the Christian Medical College, Vellore, India)	MSc Nephrology
India	1	Nephrology	Madras Medical Mission, Chennai -India	ISN Fellowship
South Africa	1	Nephrology	UCT, Groote Schuur Hospital, Cape Town –South Africa	Mphil Nephrology (UCT) ISN Fellowship, Fellowship of College of Medicine of South Africa
	1	Nephrology	Witwatersrand University, Chris Hani Baragwaneth Hospital, Johannesburg, South Africa	ISN Fellowship, Fellowship of College of Medicine of South Africa
	1	Nephrology	Stellenbosch University/ Tygerberg Hospital, South Africa	ISN Fellowship, Fellowship of College of Medicine of South Africa
	1^b^	Paediatric nephrology	University of Cape Town, Red Cross Children Hospital, South Africa	ISN/IPNA Fellowship, Post-graduate diploma, University of Cape Town
Russia^c^	2	Nephrology	Nizhny Novgorod State Medical University, Russia	MMed Nephrology
Norway	1	Nephropathology	University of Bergen/Haukeland University Hospital, Norway	ISN Fellowship

*UiB* University of Bergen, *UCT* University of Cape Town

^a^Two of the five nephrologists are from Ethiopia and Uganda

^b^MUHAS trained nephrologist

^c^Government supported training

### Advocacy for nephrology

Several advocacy campaigns for kidney health have been carried out in Tanzania largely under coordination with the Nephrology Society of Tanzania (NESOT). NESOT was established in 2012 and was then affiliated with the International Society of Nephrology in 2013 [26, 28]. Nephrologists in Tanzania have worked closely with Ministry of Health and other stakeholders in commemorating World Kidney Day annually with awareness raising and advocacy activities among the general population including screening for kidney health as well as diabetes and hypertension. Scientific conferences are conducted annually to provide a platform for continuous nephrology education and training for doctors, nurses and other stakeholders.

NESOT which has five zonal chapters in Tanzania; Eastern, Central, Lake and Southern, has provided important platform for engaging the government, health facilities, individual clinicians and other stakeholders in advancing nephrology services and improving access to this Tanzania. Increased awareness increased the number of doctors enrolling for local and international nephrology training programs. Networking among stakeholders has been fostered through various activities organized by NESOT including conferences and workshops.

### Collaborating partners for nephrology services in Tanzania

Several international institutions have supported development of nephrology in Tanzania for the last decades. These supports have benefited several hospitals and medical schools with significant impact on establishing and advancing services for Tanzania. Table 3 below describes various international collaborations with Tanzanian institutions to support growth and sustainability of nephrology services.

**Table 3:** International collaborations for supporting nephrology in Tanzania

Recipient institutions in Tanzania	Supporting institutions	Support provided
Muhimbili National Hospital (MNH)	Haukeland University Hospital/ University of Bergen, Norway	Donation of 15 dialysis machines
		Training of dialysis technicians and histopathology technician
		Donation of one GE Logic 7 Ultrasound Machine (GE Logic 7), 2 Spring Loaded Magnum Bard Biopsy Guns and several kidney biopsy needles
		Conducted ultrasound guided renal biopsy training
	Madras Medical Mission	Donation of dialysis machine for ICU
	BLK Hospital, New Delhi	Provided short time training for transplantation to urologists, nephrologist, nurses, anaesthesiologist, laboratory technicians, histotechnicians
		Supported kidney transplantation at MNH
	Sakra Hospital, Bangalore-India	Visiting transplant surgeons to support local kidney transplantation
	Saifee Hospital, Mumbai-India	Visiting transplant surgeons to support local kidney transplantation
Muhimbili University of Health and Allied Sciences (MUHAS)	University of Bergen/ Haukeland University Hospital	Training of nephrologists and supported establishment of nephrology training in Tanzania
	Christian Medical College- Vellore, Tamil Nadu India	Training of first batch of nephrologists and supported establishment of nephrology training in Tanzania
		Providing on-going external rotation training centre for MUHAS nephrology training
	African Paediatric Fellowship Program (APFP), Cape Town, South Africa	Supported paediatric nephrology training
Kilimanjaro Christian Medical Centre (KCMC)	Queen’s University, Ontario - Canada	ISN Sister Centre Program Provided visiting nephrologist, visiting dialysis nurses and funds for educational activities
	International Society of Nephrology	Training support for nephrologists
	International Society of Peritoneal Dialysis (ISPD)	Provided visiting nephrologists and training for the initiation of peritoneal dialysis
	Manchester Royal Infirmary	The Transplantation Society-ISN sister transplant centre
		Vascular access training camps
Mbeya Referral Hospital	Madaktari Afrika, Virginia, USA	Provided visiting nephrologist who provided training and support in establishing nephrology services
University of Dodoma (UDOM)	Tokushukai Medical Hospital (Tokuda Foundation)/Tokyo Women Medical University, Japan	Donation of 10 dialysis machines and dialysis training for doctors and nurses
		Training on kidney transplantation for doctors, nurses, pharmacists, immunologists and technicians
Benjamin Mkapa Hospital	Tokushukai Medical Group, Japan	Training on kidney transplantation for doctors, nurses, pharmacists, immunologists and technicians
		Supported kidney transplantation of four patients
Mnazi Mmoja Hospital, Zanzibar	University of Bergen/ Haukeland University Hospital, Norway	Donation of dialysis machines
		Dialysis training for nurses and doctors
		Visiting dialysis nurse providing training and support for nurses and doctors
		Nephrology Tripartite workshops in collaboration with MNH and Haukeland University Hospital
Nephrology Society of Tanzania (NESOT)	International Society of Nephrology (ISN)	ISN Fellowship training for nephrologists and pathologist

## Discussion

Nephrology services have seen dramatic changes in the past two decades in Tanzania. This is attributed to political will, government support, commitment of health care providers for training and increase in the awareness about the burden of kidney diseases, as well as active clinical research. Strong public-private partnership has promoted establishment of privately-owned dialysis units and this has strengthened provision of renal replacement therapy. International support and collaborations have played key roles in supporting capacity building, particularly in the training of health care providers and donation of equipment, which made introduction of new services possible. Nephrology training program in Tanzania is one of the remarkable achievements through which 57.1% (8/14) of all nephrologists in Tanzania have been trained, and the strong relationship between clinical and academic training has been of great importance [26].

Tanzania has witnessed a dramatic growth in number of dialysis units in the last decade, increasing from just one unit to 27 units in 2018 [25]. All dialysis units in Tanzania are located in 7 big cities in Tanzania, with majority of capacity in Dar es Salaam which accounts for about 10% of the Tanzanian population. There is limited access to regional dialysis services in the rest of the country as reported by Meremo et al. and Katunzi et al. in recent studies [20, 29].

The other important challenge of dialysis services is the high cost of provision of haemodialysis, which has been estimated to be more than USD 27,400 per year per patient at Muhimbili National Hospital [30]. The price for each dialysis session in Tanzania ranges from USD 120–150. Funding for dialysis in Tanzania is through National Health Insurance Fund (NHIF), initially established to cover public servants and now also enrolling members from private sector. NHIF membership is 7.1% of Tanzania population and provides full reimbursement for dialysis services for its members [31]. Tanzanians who are not covered by NHIF must pay out of pocket for dialysis services, implying that only members of this fund have guaranteed access to dialysis and other nephrology services [28, 29]. As the dialysis activity is expected to rise substantially soon reducing costs of dialysis is of paramount importance. The professional commitment of Tanzanian nephrologists and NESOT aiming at fulfilling the goals of the ISN to reduce preventable deaths from acute kidney injury in low-income countries also highlights the needs of reducing costs and implementing a nationwide peritoneal dialysis program [32].

Kidney transplantation services were initially provided overseas making Tanzania one of few countries in SSA which has supported and provided funding for this service out of the country. However, these services are currently offered in the country with strong collaborations with international institutions. Regulated travel for transplantation as well as provision of local services played a significant role in curbing transplant tourism which has been reported widely in SSA region, contributing to serious medical and psychosocial problems [33–35]. Access to transplantation services which was limited with travel for transplantation in Tanzania is expected to improve with scaling up of local transplantation program.

The local transplantation program at two hospitals in Tanzania will improve access to these services to many needy patients. There is still a lot of work which needs to be done and this includes legal support systems for organ donation as currently there is no legislature governing provision of these services. Transplantation services in Tanzania are provided with observation of Istanbul declaration on organ trafficking and transplant tourism and using special guidelines in the Muhimbili National Hospital establishment act [36].

In order to sustain and build on all these important achievement the NESOT needs to work closely with the government and all other stakeholders to promote enrolment of more nephrology trainees, speed up the establishment of renal nursing training, and improve access by encouraging establishment of dialysis units in all regions. NESOT should also promote and support establishment of peritoneal dialysis and growth of kidney transplantation program. There should be an emphasis on continuous education to improve their skills including nutrition especially for CKD patients [37], prevention and early identification of patients with kidney diseases.

NESOT should also promote collaboration with the government of Tanzania and other stakeholders to prepare laws and policies to provide legal framework for supporting kidney transplantation and other services. These policies should also guide all stakeholders to increase focus on prevention of kidney diseases focusing on modifiable risk factors and outreach renal services.

## Conclusion

There has been significant improvement of nephrology services in Tanzania with establishment of local nephrology training programs and kidney transplantation services. The long-standing international collaboration and support have been instrumental in these developments and milestones. These collaborative efforts and achievements should be used as good model for supporting nephrology services in other low-income countries, especially in the African region. To meet the increased need of treating patients with ESKD it is of paramount importance to reduce the costs of dialysis. ISN, IPNA and International Society of Peritoneal Dialysis (ISPD) should continue their efforts in capacity building for nephrology care by continuing to establish programs to support nephrology training in countries with similar resource limitations as Tanzania. National nephrology societies play important role in establishing and strengthening nephrology services through advocacy, linkage between governments and international professional societies like ISN, IPNA and ISPD. Therefore, there should be efforts to support creating of these societies in lower and middle-income countries.

## Data Availability

The datasets used for this work can be requested from the corresponding author on reasonable request.

## Abbreviations

AKI: Acute Kidney Injury
ANIO: American Nephrologists of Indian Origin
BMH: Benjamin Mkapa Hospital
CMC: Christian Medical College
CNC: Clinical Nephropathology Certificate
eGFR: estimated Glomerular Filtration Rate
ESKD: End Stage Kidney Disease
HIV: Human Immunodeficiency Virus
IPNA: International Paediatric Nephrology Association
ISN: International Society of Nephrology
ISPD: International Society of Peritoneal Dialysis
KCMC: Kilimanjaro Christian Medical Centre
LMIC: Lower- and Middle-Income Countries
MNH: Muhimbili National Hospital
MUHAS: Muhimbili University of Health and Allied Sciences
NCD: Non-Communicable Disease
NESOT: Nephrology Society of Tanzania
NHIF: National Health Insurance Fund
SSA: Sub Saharan Africa
SYL: Saving Young Lives
UCT: University of Cape Town
UiB: University of Bergen
USD: United State Dollar
